# Effects of Vitamin D_2_ and 25-Hydroxyvitamin D_2_ Supplementation on Plasma Vitamin D Epimeric Metabolites in Adult Cats

**DOI:** 10.3389/fvets.2021.654629

**Published:** 2021-06-07

**Authors:** Catherine E. Ruggiero, Robert C. Backus

**Affiliations:** Department of Veterinary Medicine and Surgery, College of Veterinary Medicine, University of Missouri, Columbia, MO, United States

**Keywords:** feline, C-3 epimer, cholecalciferol, ergocalciferol, 24,25-dihydroxyvitamin D, 3-epi-24,25(OH)_2_D_2_,1,25(OH)D_2_, calcitriol

## Abstract

Feline vitamin D status is based on dietary consumption but metabolism of this essential nutrient and the efficacy of supplementation forms are poorly described in cats. The aim of this study was to further elucidate the metabolites of vitamin D_2_ in cats and to compare the effectiveness of vitamin D_2_ and 25(OH)D_2_ for increasing feline vitamin D status. Eight adult male castrated domestic shorthair cats received vitamin D_2_ or 25(OH)D_2_ in a single crossover design. Vitamin D_2_ was dosed daily in a molar equivalent dosage to vitamin D_3_ ingested in the diet while 25(OH)D_2_ was provided at a daily dose of 20% molar equivalent intake of dietary vitamin D_3_ based on its expected higher potency. Plasma concentrations of 25-hydroxyvitamin D epimers were evaluated at baseline then every 2 weeks for a total of 10 weeks. Analysis of multiple vitamin D metabolite concentrations was completed at the end of each supplementation period, followed by a washout period preceding the second phase of the crossover trial. Results showed that supplementation with 25(OH)D_2_ more effectively and rapidly raised circulating 25(OH)D_2_ levels in cat plasma compared to vitamin D_2_. Formation of C-3 epimers of 25(OH)D_3_, 25(OH)D_2_, and 24,25*R*(OH)_2_D_3_, but not 24,25(OH)_2_D_2_, were observed in feline plasma. The abundant concentrations of epimeric forms of vitamin D metabolites found in circulation suggest that these metabolites should be considered during vitamin D analyses in cats. Further studies using 25(OH)D and vitamin D_2_ forms are needed to conclude safety and efficacy of these vitamers for supplementation in this species.

## Introduction

Unlike some mammals, cats cannot synthesize adequate vitamin D from UV light and therefore have a dietary requirement for this nutrient (1). Cholecalciferol (vitamin D_3_) is found in animal tissues and is provided in most commercial feline diets through ingredients or supplementation (2). Ergocalciferol (vitamin D_2_) is a fungal and plant source of vitamin D (3) that is currently found in feline supplements for homemade diets. This may offer an alternative for cats that must avoid specific animal protein sources due to adverse food reactions or food allergies. Additionally, vitamin D_2_ is less likely to induce toxicity in cats when provided at an equivalent dose to D_3_ (4). However, knowledge regarding vitamin D metabolism, including utilization of vitamin D_2_, in cats is extremely limited.

Ingested vitamin D is absorbed from the diet via enterocytes, incorporated into plasma lipoproteins, and transported to the liver by vitamin D binding protein, where it is stored and hydroxylated to 25(OH)D (5–7). This metabolite is then transported to the kidney where it is hydroxylated again at position 1 of the A-ring to form the hormone 1,25(OH)_2_D, or calcitriol (8). Together with other metabolites such as 24,25(OH)_2_D and 1,24,25-dihydroxyvitamin D (1,24,25(OH)_2_D), this hormone plays an important role in calcium homeostasis, skeletal health, and possibly other physiological functions (9, 10). Epimeric forms of many of these vitamers have been identified in multiple species, with an epimer at position 3 of the A-ring of 25-hydroxyvitamin D_3_ (3-epi-25(OH)D_3_) recently identified in cats (11). The function of C-3 epimers of vitamin D metabolites is unknown, but they appear to be endogenously produced and biologically active metabolites (12–17).

Since all vitamin D is essentially converted to 25(OH)D and this metabolite is found abundantly in circulation, it is the most commonly measured indicator of vitamin D status (18). Circulating 25(OH)D_3_ is an active metabolite and has been found to be more potent at influencing vitamin D status than vitamin D_3_ when administered as a supplement to dogs (19, 20), rats (21, 22), chicks (21), and humans (23, 24). In fact, 25(OH)D_3_ is reported to be up to 10 times more potent than vitamin D_3_ in humans (23) and may prove an effective form of supplementation for people and animals in diseases such as chronic kidney disease and cardiovascular disease (23, 25, 26).

Vitamin D supplementation is common in humans, and both cholecalciferol and ergocalciferol are used. With increasing recognition of vitamin D status in companion animals, supplementation may become more common in the future and supplement form will need to be considered (2). Although research in cats is limited, one study suggests a lower bioavailability of vitamin D_2_ compared to D_3_ in felines (27). Cats are not the only species that appear to discriminate against vitamin D_2_; a similar pattern is observed in pigs (28), chicks (28, 29), horses (30), and monkeys (31, 32). Rats, on the other hand, exhibit a preference for vitamin D_2_ (28, 33). In humans, vitamins D_2_ and D_3_ have generally been considered equipotent, though a recent meta-analysis suggests a superiority of D_3_ in raising 25(OH)D concentrations compared to D_2_ (34). Numerous explanations for the increased potency of D_3_ have been suggested, including greater affinity for hydroxylase enzymes and vitamin D receptors (32, 35–37). Most of the vitamin D_3_ metabolites have a D_2_ counterpart, suggesting a similar metabolic pathway based on 25-hydroyxlation (38). Additionally, a unique 24-hydroxylation pathway occurs in vitamin D_2_ metabolism to form metabolites (24(OH)D_2_, 1,24(OH)_2_D_2_, 1,24,25(OH)_3_D_2_), that appear less potent than those derived from vitamin D_3_, which may lead to more rapid deactivation of vitamin D_2_ (39–44).

Given the paucity of information surrounding vitamin D metabolism in cats in general, and the unknown effectiveness of vitamin D_2_ supplementation in cats specifically, the purpose of this study was to investigate vitamin D metabolism when cats are supplemented vitamin D_2_ or 25(OH)D_2_ in addition to vitamin D_3_ found in the diet. As vitamin D_2_ is derived from non-animal sources, its use in cats as a supplement in place of vitamin D_3_ is hypothetically advantageous in cases of suspected allergic reaction to food of animal origin. In many such cases, a tolerated meat, poultry, or fish source is empirically identified and in turn used as a dietary ingredient, though it may contain vitamin D_3_ and thereby provide a background amount of vitamin D_3_ (45). Impetus for study of 25(OH)D_2_ relates to reputed superior bioavailability of 25(OH)D compared to vitamin D (46). Supplementation with 25(OH)D might correct vitamin D deficiency when dietary vitamin D is ineffective, as in cases of inflammatory bowel disease, which are associated with vitamin D deficiency, and may occur secondary to allergic intolerance of food (47). Specifically, the aims of this study were: (1) to identify vitamin D metabolites present in cats, including epimeric forms of vitamin D_2_ and D_3_ metabolites that have been previously identified in other species, and (2) to compare the effectiveness of vitamin D_2_ and 25(OH)D_2_ for increasing vitamin D status in cats. A difference in effectiveness was hypothesized based on reports in other species of greater potency of 25(OH)D_3_ compared to vitamin D_3_.

## Materials and Methods

### Animals

Eight university-owned, purpose-bred male castrated domestic shorthair cats were included in the study. The median age of cats was 7.5 years (range 7–12 years) with median body weight of 4.7 kg (range 4.2–5.9 kg). Cats were considered healthy based on physical examination, activity, and results of complete blood count, plasma biochemistry, and total T4 concentrations evaluated immediately prior to enrollment. Cats were housed in an American Association for Laboratory Animal Science accredited, temperature, humidity, and light cycle-controlled facility. They were individually housed for ~8 h during the day to monitor food intake, then group-housed in two separate groups for the remainder of the day. Food was provided once daily in the morning (typically 7:30–9:30); water was available at all times. Prior to beginning and every 2 weeks during the study, each cat's body weight was recorded using the same scale immediately before feeding. Body condition scoring using a 9-point scale (48) was also performed. Venous blood was then collected by jugular venipuncture and plasma was collected every 2 weeks for vitamin D metabolite determination. Biochemical analyses, including complete blood count and clinical biochemistry, were performed prior to and at the end of the experiment. Care of animals followed National Institutes of Health Guide for the Care and Use of Laboratory Animals (49) and was approved by the Animal Care and Use Committee of the University of Missouri (Protocol 9214).

### Diet

The cats were maintained exclusively on the same commercial dry extruded feline diet (Supplementary Table 1) at an energy intake known to maintain body weight for each individual (48–61 g/d; 877–1,110 kJ/d). This diet was fed for several months prior to initiation of the study and was readily accepted by the cats. Consumption of the offered amount was recorded daily in grams. As indicated by its label claim, the diet passed Association of American Feed Control Officials (AAFCO) feeding trials for maintenance of adult cats. A single batch of diet was fed throughout the duration of the experiment. Vitamin D_3_ and D_2_ concentrations in the study batch were measured by an external laboratory (Eurofins Nutrition Analysis Center, Des Moines, IA, USA). Vitamin D_3_ content was 318 IU/100 g with no detectable (<4 IU/100 g) vitamin D_2_.

### Experimental Design

A single crossover design was used. Firstly, the cats were systematically assigned to one of two treatment groups. For this, the cats were ranked based on dietary intake of vitamin D_3_ then assigned to the groups so that mean vitamin D_3_ intakes between the groups were similar. Four cats initially received vitamin D_2_ (Sigma-Aldrich, St. Louis, MO, USA) daily in a molar equivalent dosage to the vitamin D_3_ they were ingesting from the diet based on commercial analysis of the diet and daily intake (0.80–0.86 μg/kg body weight per d). The other four cats initially received 25(OH)D_2_ (Isosciences, Ambler, PA, USA) daily at a dosage that was equal to 20% of the daily molar equivalent intake of dietary vitamin D_3_ (0.16–0.18 μg/kg body weight per d). Selected dosage of 25(OH)D_2_ was based on data obtained by one of the investigators (RCB) in a prior canine study, indicating an ~5-times greater oral potency of 25(OH)D_3_ relative to vitamin D_3_ for affecting change in plasma 25(OH)D_3_ concentration (50). In this study, extraordinarily high plasma concentrations of 25(OH)D_3_ resulted after only 2 weeks of dosing of 25(OH)D_3_ at 2.3 μg/kg^0.75^. Due to concern about potential hypervitaminosis D, potency of 25(OH)D_3_ relative to vitamin D_3_ in this work was not estimated from equilibration concentrations of 25(OH)D_3_ in plasma. Rather, the relative potency was estimated from differences in rate of change in plasma 25(OH)D_3_ concentration from baseline values. For dosing of 25(OH)D_2_ in the present study, ethanol was used for dilution of stock solutions of vitamin D_2_ and 25(OH)D_2_ to 0.5 and 0.1 μg/μL, respectively. Each day, a small volume (7–10 μL) of ethanolic stock solution was applied to a single kibble of diet mixed in with the meal for voluntary consumption. Plasma concentrations of 25(OH)D_3_ and its C-3 epimer, as well as 25(OH)D_2_ and its C-3 epimer, were evaluated initially and then every 2 weeks. Following observation of an apparent plateau in plasma concentration of 25(OH)D_2_ among the cats after 10 weeks, analyses of multiple vitamin D metabolite concentrations were completed. Thereafter, the D_2_ vitamer supplementations were discontinued for a washout period of 12 weeks prior to beginning the second phase of the crossover. Length of the washout period was determined by monitoring plasma concentrations of 25(OH)D every 2 weeks until the concentrations of 25(OH)D_2_ were below the level of quantification. Limits of quantification and detection were defined as follows: peaks of UV absorbance recorded during reverse-phase HPLC were quantified if they coincided with the retention time of D metabolite standard and their amplitude was 5-times greater than the amplitude of the standard deviation of background noise. Metabolites were considered detected when a peak coincided with retention time of standard and amplitude was 2-times greater than standard deviation of background noise. Limits of quantification for 25(OH)D_3_, 3-epi-25(OH)D_3_, 25(OH)D_2_, 3-epi-25(OH)D_2_, 24,25(OH)_2_D_3_, 3-epi-24,25(OH)_2_D_3_, and 24,25(OH)_2_D_2_ were determined to be 3.0, 2.0, 3.0, 3.8, 4.7, 10.1, and 9.1 ng/mL, respectively.

### Laboratory Analyses

Clinical hematology (Sysmex xT-2000i; Sysmex, America, Inc.) and plasma chemistry analyses (Beckman AU480; Beckman Coulter, Inc.) were performed at the University of Missouri Veterinary Medical Diagnostic Laboratory, Columbia, MO prior to and at the end of each 10-week supplementation period. Clinical hematology and plasma chemistry were analyzed immediately after each supplementation period to confirm there were no adverse effects of supplementation. Plasma was also stored at −20°C for repeat chemistry analysis at the end of the trial to reduce inter-assay variation. Serum was reserved after each supplementation period and stored at −20°C, then analyzed for parathyroid hormone (PTH) and ionized calcium concentrations at an external laboratory (Michigan State University Veterinary Diagnostic Laboratory, East Lansing, MI, USA) upon conclusion of the study.

### Plasma 25(OH)D Analyses

For monitoring of vitamin D status every 2 weeks, plasma concentrations of 25(OH)D_3_ and 25(OH)D_2_ and their respective C-3 epimers were determined using extraction and HPLC methods of Lensmeyer and colleagues (51) with modifications. The modifications were required because quantification of some metabolites was precluded by the overlap of HPLC peaks of 3-epi-25(OH)D_3_ and 25(OH)D_2_, as reported elsewhere (52). For the determinations, 0.5 mL of plasma was incubated overnight (14–16 h) with internal standard, tritiated 25(OH)D_3_ (25-[26,27-^3^H]-hydroxyvitamin D_3_) (Perkin Elmer, Boston, MA, USA). The plasma was thereafter combined with 1.0 mL acetonitrile (CH_3_CN), incubated for two 5 min periods, twice vortexed, and for 10 min centrifuged at 2,000 × g at ambient temperature (22–24°C). The supernatant was diluted with 0.5 mL water and loaded on a conditioned solid-phase extraction column (Strata^TM^-X, 33 μm, 60 mg, Phenomenex, Torrance, CA, USA). The column was washed with 2 mL CH_3_CN-water (35:65, v/v) and eluted with 2 mL CH_3_CN. The eluent was dried by centrifugal evaporation for 75 min. Eluent residue was reconstituted with 250 μL hexanes-2-propanol (94:6, v/v). A portion of the reconstitute (200 μL) was injected on an equilibrated (2.0 mL/min, hexanes-2-propanol, 94:6, v/v) normal-phase column (4.6 x 250 cm, Zorbax Sil, Agilent Technologies, Santa Clara, CA, USA). Eluted fractions containing metabolites of 25(OH)D_2_ and 25(OH)D_3_ were dried and then reconstituted with 100 μL methanol and diluted with 50 μL water as needed for subsequent quantification by reverse-phase HPLC.

A portion (120 μL) of the reconstituted normal-phase fractions containing 25(OH)D_3_ metabolites was injected on to a heated (50°C), reverse-phase HPLC column (Zorbax SB-CN, Agilent Technologies, Santa Clara, CA, USA), equilibrated with methanol-water (67:33, v/v), flowing at 1.2 mL/min, as described by Lensmeyer et al. (51). Eluting 25(OH)D_3_ and 3-epi-25(OH)D_3_ were quantified from area-under-the-curve (AUC) of peak UV absorbance at 265 nm. Eluting 25-[26,27-^3^H]-hydroxyvitamin D_3_ was collected, dried, and quantified by liquid scintillation counting. Reconstitute of the normal-phase fraction containing 25(OH)D_2_ metabolites was spiked with internal standard (25 ng of 25(OH)D_3_) and a portion of it (120 μL) injected on the reverse-phase column for quantification of 25(OH)D_2_, 3-epi-25(OH)D_2_, and internal standard.

For calculation of the plasma metabolite concentrations, recovery of all the metabolites was assumed to be in equivalent proportion to recovery of tritiated internal standard, 25-[26,27-^3^H]-hydroxyvitamin D_3_. This assumption was verified for 25(OH)D_2_ and 3-epi-25(OH)D_2_ in pooled plasma collected from cats not consuming dietary vitamin D_2_. For this, plasma aliquots (0.5 mL, *n* = 5) were spiked with 25 ng of 25(OH)D_2_, 25 ng of 3-epi-25(OH)D_2_, and 1,200 dpm of 25-[26,27-^3^H]-hydroxyvitamin D_3_. Mean (± standard deviation) recoveries of the metabolites were determined to be 43.1 ± 5.9, 44.0 ± 2.6, and 47.3 ± 2.6%, respectively.

### Mono- and Di-Hydroxyvitamin D Metabolites

Plasma collected during the last week of each supplementation period was additionally analyzed for di-hydroxyvitamin D metabolites. For this, 1.0 mL of plasma was extracted by methods of Hollis (53) following overnight incubations with methanolic solutions (10 μL) of internal standards: 25-[26,27-^3^H]-hydroxyvitamin D_3_ and 1α,25-[26,27-^3^H]-dihydroxyvitamin D_3_ (Perkin Elmer Boston, MA, USA). Samples were vortex-mixed for 20 s in 1.0 mL CH_3_CN and centrifuged (2,000 × g) for 20 min at ambient temperature. Resulting supernatant was vortex-mixed with 1.0 mL water and applied to a conditioned solid-phase column (Bond Elut C18; Agilent Technologies, Santa Clara, CA, USA). The column was washed with water (5 mL) and methanol-water (70:30, v/v; 5 mL), then air dried for 10 min. The column was eluted with hexanes-methylene chloride (90:10, v/v; 5 mL) and then hexanes-2-propanol (95:5, v/v; 5 mL). Residue of eluents was reconstituted and fractionated by the above described normal-phase HPLC method with two minor variations: The mobile-phase was hexanes-2-propanol (96:4,v/v) and six fractions were collected, each fraction coinciding with the retention time of standards of vitamin D_2_ and D_3_ forms of 25(OH)D, 24,25(OH)_2_D, and 1,25(OH)_2_D. The fractions were assumed to contain both C-3 epimers because little difference was observed between retention times of epimeric standards that were commercially available, i.e., 3-epi-25(OH)D_3_, 3-epi-25(OH)D_2_, and 3-epi-24*R*,25(OH)_2_D_3_ (Isosciences, Ambler, PA, USA).

The above described reverse-phase HPLC method was used to quantify both epimeric forms of 25(OH)D_3_, 25(OH)D_2_, 24,25*R*(OH)_2_D_3_ and 24,25(OH)_2_D_2_. The method was slightly modified for quantification of the 24,25(OH)_2_D epimers: Internal standard, 25 ng of 25(OH)D_3_, was added to the 24,25(OH)_2_D reconstitutes prior to HPLC quantitation. The HPLC mobile-phase was changed to a gradient in which the methanol-water proportion was increased after 20 min from 60:40 (v/v) to 70:30 (v/v) for elution of internal standard, 25(OH)D_3_. Plasma concentrations of the metabolites were calculated the same as described above for concentrations of the 25(OH)D_3_ and 25(OH)D_2_ metabolites.

The normal-phase fraction containing 1,25(OH)_2_D_2_ and that containing 1,25(OH)_2_D_3_ and 1α,25-[26,27-^3^H]-dihydroxyvitamin D_3_ were each aliquoted into thirds. One third of the fraction containing 1,25(OH)_2_D_3_ was dried and radioactivity in residue determined by liquid scintillation counting. The remaining aliquots of the fractions were added to borosilicate glass tubes (12 x 75 mm) to which 10 μL methanol was also added. The glass tube contents were dried and the amount of 1,25(OH)_2_D_3_ or 1,25(OH)_2_D_2_ in residue of the fractions was determined using a commercially available RIA kit (1,25-Dihydroxy Vitamin D RIA, Immunodiagnostic Systems, Ltd., Boldon, Tyne, & Wear, UK). A procedural deviation in use of the RIA kit was that standards were borosilicate glass tubes that contained dried residues of 10 μL aliquots of methanolic dilutions (0.4, 1.6, 6.3, and 25 pg/mL) of 1,25(OH)_2_D_3_ (Isosciences, Ambler, PA, USA) and organic mobile phase (hexanes-2-propanol, 96:4, v/v) of a volume equivalent to that aliquoted for RIA of samples (~1.5 mL). Another variation in procedure in use of the RIA kit was that incubation of tubes with radiolabel was extended from 1 to 3 h before separation of bound from free radiolabel. The response in inhibition of binding was linear with natural log of mass of 1,25(OH)_2_D_3_ standard in tubes [bound % = −14 × ln (1,25(OH)_2_D_3_ pg) + 92; *r* = 0.998]. The amount of 1,25(OH)_2_D_3_ calculated to affect a 50% binding was 19 pg.

The plasma concentrations of vitamin D metabolites were determined using the above methods in 1.0 mL replicates (*n* = 6) of pooled plasma from cats receiving no dietary vitamin D_2_. The plasma replicates were spiked with 50 ng each of 25(OH)D_2_, 3-epi-25(OH)D_2_, and 24*RS*,25(OH)_2_D_2_ (Isosciences, Ambler, PA, USA) prior to analysis. Mean (± standard deviation) recoveries of the metabolites were found to be 41.4 ± 4.4, 44.3 ± 6.2, and 54.2 ± 18.7%, respectively. Recoveries of the tritiated labels of 25(OH)D_3_ and 1,25(OH)_2_D_2_ were determined to be 44.8 ± 2.7 and 74.9 ± 13.2%, respectively. Coefficients of variation determined for 25(OH)D_2_, 3-epi-25(OH)D_2_, and 24*RS*,25(OH)_2_D_2_ were 10.7, 13.9, and 22.9%, respectively.

### Metabolite Identity Confirmation

The LC-MS/MS methods of Kaufmann et al. (54) were used with modifications to confirm that UV absorbance of vitamin D metabolites were causal of reverse-phase HPLC peaks recorded during retention times of metabolite standards. Initially, HPLC eluent was collected during periods (~1.5 min) when UV peaks of supposed metabolite were observed. The eluent fractions from each cat were combined across cats according to metabolite fraction. Metabolite in the pooled fractions was extracted into chloroform by methods of Bligh and Dyer (55). For this, water and chloroform were added to the fractions in amounts sufficient to form a mixture of 0.9:1.0:1.0 (v/v/v) water-chloroform-methanol. The mixture was vigorously shaken for 1 min and thereafter allowed to stand until development of a chloroform layer, which was then removed and dried. Metabolite in residue of the chloroform extract was derivatized in a 1.0 mL glass vial by incubation for 30 min with freshly prepared labeling solution – 25 μL of briefly sonicated, dry, methylene chloride containing 0.1 mg/mL of 4-[2-(6,7-dimethoxy-4-methyl-3,4-dihydroquinoxalinyl-)ethyl]-1,2,4-triazoline-3,5-dione (Key Synthesis, Oaks, PA, USA). The incubation was extended another 60 min after addition of another 25 μL of labeling solution. The derivation was stopped by addition of 40 μL ethanol. After drying, derivatized metabolites were dissolved in 250 μL methanol-water (60:40, v/v) and quantified by the LC-MS methods of Kaufman et al. (54) by the University of Missouri, Charles W. Gehrke Proteomics Center.

### Statistical Analyses

Statistical analysis was performed with statistical software [SAS 9.4, SAS Institute, Cary, NC, USA; Microsoft Excel (2016), Microsoft Corporation, Redmond, WA, USA]. Variable observations were accepted as normal if means and medians were similar and skew and excess kurtosis were both between −1.0 and 1.0. Plasma 25(OH)D_3_ and 3-epi-25(OH)D_3_ concentrations and anion gap, serum PTH concentration, and blood monocyte count were observations normalized by logarithmic transformation.

Repeated-measures, mixed-model ANOVA was used to determine the significance of effect of treatment (vitamin D_2_ vs. 25(OH)D_2_) on plasma concentrations of 25(OH)D_3_ and 25(OH)D_2_ and their C-3 epimers. Fixed effects were D_2_ vitamer type, crossover phase, and their interaction while random effects were subjects (cats). Significance of differences of sampling week were identified using Tukey-adjusted, multiple comparisons. Significance of effect of oral D_2_ vitamer form on plasma concentrations of dihydroxyvitamin D metabolites [24,25(OH)_2_D_2_, 24,25(OH)_2_D_3_, 1,25(OH)_2_D_2_, 1,25(OH)_2_D_3_] were determined with Wilcoxon two-sample tests comparing the group receiving vitamin D_2_ first to the group receiving 25(OH)D_2_ first. For single repeated measures, paired-*t* tests were used to test significance of oral D_2_ vitamer type [D_2_ vs. 25(OH)D_2_] on normal observations. Sign-rank tests were used to test significance of single repeated, non-normal observations (i.e., serum ionized calcium concentration, blood basophil count, and plasma concentrations of phosphorus, urea nitrogen, creatinine, chloride, albumin, and total protein).

The number of cats selected for study was based on a power analysis using reported mean and variance observations on peak plasma 25(OH)D_2_ concentrations in 10 adult cats given oral boluses of vitamin D_2_ (27). The analysis indicated that paired observations of 25(OH)D_2_ concentrations in seven cats should be sufficient for detecting a 50% mean difference caused by a treatment effect with α = 0.05 with a power of 80%.

Central tendency and dispersion of non-normal observations are reported as median and range, respectively. Results for normal observations are reported as mean and SEM. Effects were considered significant if *P* < 0.05.

## Results

### Animals

A total of eight cats were initially included in the study. All diet presented each day was typically consumed. The median (range) energy intake for cats at baseline was 79.8 (73.2–82.4) kcal/kg^0.67^. Cats received dietary vitamin D_3_ in their commercial diet (0.65–0.95 μg/kg body weight per d). Prior to starting the D_2_ vitamer supplementations, body condition score (BCS) of the cats was ideal (BCS 5/9, 20% body fat, *n* = 3) or slightly above ideal condition (BCS 6/9, ~ 25 to 30% body fat, *n* = 5). Food presentation was adjusted by 5–10% to maintain/achieve ideal BCS throughout the study. While vitamin D_3_ intake varied slightly with adjustments to food intake, this did not appear to have a substantive effect on D status as there was no significant crossover phase effect on plasma 25(OH)D_3_ concentrations.

One cat was euthanized after the first phase of the crossover and therefore excluded from statistical analysis. During the washout period following 10 weeks of supplementation with 25(OH)D_2_, this cat was found to have an increase in plasma urea nitrogen (from 34 to 40 mg/dL, reference interval 19–35 mg/dL), while plasma creatinine was unchanged from the pre-trial value (1.4 mg/dL) and within the reference interval (0.9–2.0 mg/dL). The concentrations of the total of 25(OH)D_3_ and 25(OH)D_2_ at this time point were 44.8 ng/mL for the beta epimers and 13.1 ng/mL for alpha C-3 epimers, respectively. During the 10 weeks of supplementation, the mean (range) plasma concentrations of the of total 25(OH)D for this cat were 47.3 (44.8–70.1) ng/mL for beta epimers and 15.0 (9.1–21.8) ng/mL for alpha epimers. These values were similar to plasma concentrations observed in other cats in the study who received 25(OH)D_2_ supplementation (Table 1). This cat was later euthanized during the washout period, 11 weeks after discontinuing 25(OH)D_2_ supplementation, following 1 week of clinical signs (anorexia, vomiting, lethargy) and the development of azotemia (BUN 163 mg/dL, creatinine 6.0 mg/dL) and other plasma biochemistry changes consistent with renal failure. Abnormal necropsy findings included asymmetric and overall diminished renal mass with left nephrolithiasis. Histopathological findings were lymphocytic interstitial nephritis, with the smaller, left kidney having diffuse interstitial, cortical fibrosis between dilated medullary and cortical tubules, and the larger, right kidney having similar pathological changes but with adjacent and greater regions of normal cortex with tubular vacuolation and minimal interstitial lymphocytic inflammation.

**Table 1:** Mean and SEM values of plasma concentrations (ng/mL) of 25(OH)D_3_ and 25(OH)D_2_ and their epimers in seven cats during 10 weeks of oral supplementation with either vitamin D_2_ or 25(OH)D_2_ while consuming a commercial feline diet containing vitamin D_3_.

	**Supplement (S)**			**Week (W)**							***P*****-value**		
**Metabolite**	**D_**2**_**	**25(OH)D_**2**_**	**SEM**	**0**	**2**	**4**	**6**	**8**	**10**	**SEM**	**S**	**W**	**S × W^‡^**
25(OH)D_3_	26.8	32.1	1.1	36.2	31.8	28.9	27.7	27.3	25.5	1.1	<0.01	<0.01	<0.01
25(OH)D_2_	13.0	25.5	2.3	0.0^*^	16.4^*^	21.0^*^	23.4	28.4	26.4	2.6	<0.01	<0.01	<0.01
Total 25(OH)D	39.5	56.0	1.1	36.2	47.7	47.6	50.0	53.3	49.4	1.1	<0.01	<0.01	<0.01
3-epi-25(OH)D_3_	14.0	15.1	1.1	12.4^a^	13.2^a,b^	13.9^b^	15.1^b,c^	16.8^c^	16.5^c^	1.1	0.08	<0.01	0.23
3-epi-25(OH)D_2_	6.7	5.3	1.0	0.0^a,^ ^†^	7.0^b,^ ^†^	6.4^b,^ ^†^	6.8^b,^ ^†^	8.2^b,^ ^†^	7.6^b,^ ^†^	1.2	0.06	<0.01	0.79
Total 3-epi-25(OH)D	20.3	20.1	1.1	12.4^a^	20.0^b^	20.3^b^	21.8^b,c^	25.4^c^	24.3^b,c^	1.1	0.78	<0.01	0.56

*The listed P-values indicate significance of effect of supplement form on metabolite concentration for the period of oral supplementation. Mean metabolite values with superscript letters indicate significance of differences of values between experimental weeks during the supplementations; values that do not have symbols in common differ (P < 0.05)*.

* *Less than 25(OH)D_3_ value for the same week (P < 0.05)*.

† *Less than 3-epi-25(OH)D_3_ value for the same week (P < 0.05)*.

‡ *Significance of Supplement-Week interaction; see Supplementary Figures 1, 2 for plots of metabolite concentrations where the interaction was significant*.

After conclusion of the second phase of the crossover, one cat experienced syncopal episodes attributed to paroxysmal third-degree AV block and persistent left bundle branch block. The cat had received supplementation with 25(OH)D_2_ during the second phase of the trial. An echocardiogram revealed mild dilation of the right atrium and ventricle. Blood glucose, cardiac troponin, ionized calcium, and other clinical hematological and biochemistry analyses repeated at the time were within laboratory reference intervals. Echocardiograms were subsequently performed on all other cats which had received 25(OH)D_2_ supplementation during the second phase (*n* = 3) and the findings were unremarkable. Serial 24-h evaluations with a Holter monitor over the next 12 weeks indicated significant improvement in conduction abnormalities in the affected cat. Rapid and complete resolution of clinical signs was observed with no further abnormalities appreciated over the next several months. Results from this cat were included in the analysis for a total of seven cats in the study.

### Effects of D_2_ Supplementation on 25(OH)D Concentrations

Plasma concentrations of 25(OH)D_2_ increased over time with both D_2_ vitamer treatments during the 10 weeks of supplementation. There was no detectable 25(OH)D_2_ in any plasma sample initially (week 0). Following supplementation with D_2_ vitamers, 25(OH)D_2_ concentrations gradually increased to plateaus by week 6. Supplementation with 25(OH)D_2_ was associated with a more rapid and greater rise in plasma 25(OH)D_2_ concentration compared to treatment with vitamin D_2_ (*P* < 0.01; Table 1).

Following supplementation with vitamin D_2_, plasma concentrations of 25(OH)D_3_ declined over time and were significantly reduced from baseline by week 8 (*P* < 0.02). Plasma concentrations of 25(OH)D_3_ were greater than those of 25(OH)D_2_ until week 6 (*P* < 0.01), after which the concentrations of 25(OH)D_3_ and 25(OH)D_2_ were not statistically different. The combined concentrations of 25(OH)D_2_ and 25(OH)D_3_ [total 25(OH)D] were unchanged by vitamin D_2_ supplementation (Table 1; Supplementary Figure 1). On the contrary, supplementation with 25(OH)D_2_ did not affect 25(OH)D_3_ concentrations but did result in an increase in total 25(OH)D by week 2 of supplementation (*P* < 0.01). The concentrations of 25(OH)D_2_ and 25(OH)D_3_ were not different at any time point other than week 0, when 25(OH)D_2_ concentrations were below 25(OH)D_3_ (*P* < 0.01; Supplementary Figure 2).

### Effects of D_2_ Supplementation on Alpha C-3 Epimeric Forms of 25(OH)D

Peaks of UV absorbance were observed coinciding with the retention time of 3-epi-25(OH)D_2_ standard in reverse-phase HPLC of extracted and fractionated plasma from all cats. Elution of the epimer at the time of the UV peaks was confirmed by the LC-MS/MS analysis described in the methods.

Similar to initial 25(OH)D_2_ concentrations, there was no detectable 3-epi-25(OH)D_2_ in any plasma sample initially (week 0). With both D_2_ vitamer supplementations, 3-epi-25(OH)D_2_ concentrations increased by week 2 (*P* < 0.03), after which no significant changes were observed (Table 1). There was no effect of D_2_ supplementation form [vitamin D_2_ vs. 25(OH)D_2_] on plasma concentrations of 3-epi-25(OH)D_2_.

Plasma concentrations of the alpha C-3 epimers remained below concentrations of their respective beta epimers [i.e., 25(OH)D_2_ and 25(OH)D_3_] at all time points. By week 10 of the D_2_ vitamer supplementations, mean concentration of 3-epi-25(OH)D_2_ was less (*P* < 0.01) and 29% of the concentration of 25(OH)D_2_ and 3-epi-25(OH)D_3_ was less (*P* < 0.01) and 65% of the concentration of 25(OH)D_3_ (Table 1). The C-3 epimer of 25(OH)D_3_ was present in higher concentrations than 3-epi-25(OH)D_2_ at all weeks during both vitamer supplementations (*P* < 0.02). After week 0, there were no significant changes in and differences between concentrations of 3-epi-25(OH)D_2_ and 3-epi-25(OH)D_3._ There was, however, a difference between the alpha epimer concentrations of 25(OH)D_2_ and 25(OH)D_3_ at weeks 8 and 10 when cats received 25(OH)D_2_ supplementation (*P* < 0.01). The total 3-epi-25(OH)D concentrations remained unchanged after week 0 during both D_2_ vitamer supplementations (Table 1).

### Effects of D_2_ Supplementation on 24,25(OH)_2_D and 1,25(OH)_2_D Metabolites

At the end of supplementations, analysis of week 10 plasma from both crossover phases was conducted to determine concentrations of dihydroxyvitamin D metabolites, including the D_3_ and D_2_ forms of 1,25(OH)_2_D, as well as 24,25(OH)_2_D and its putative C-3 epimers. Due to an analysis error that caused sample loss, concentrations of these metabolites could not be reliably determined for week 10 of the second crossover phase. Results of analyses of samples of the first phase of the crossover trial are reported here when cats received oral supplementation with vitamin D_2_ (*n* = 4) and 25(OH)D_2_ (*n* = 3) (Table 2).

**Table 2:** Median and range values of plasma vitamin D metabolites in cats after 10 weeks of oral supplementation with vitamin D_2_ (*n* = 4) or 25(OH)D_2_ (*n* = 3) while consuming a commercial feline diet containing vitamin D_3_.

	**Supplementation form**				
	**Vitamin D**_****2****_		**25(OH)D**_****2****_		***P*-value**
**Metabolite**	**Median**	**Range**	**Median**	**Range**	
1,25(OH)_2_D_3_ (pg/mL)	37.2	17.9–55.8	28.4	23.6–29.9	0.86
1,25(OH)_2_D_2_ (pg/mL)	28.4	23.6–29.9	13.5	10.7–16.0	0.71
24,25(OH)_2_D_3_ (ng/mL)	15.3	9.3–25.8	17.3	16.5–17.8	1.00
24,25(OH)_2_D_2_ (ng/mL)	nd^*^	nd	nd	nd	–
3-epi-24,25*R*(OH)_2_D_3_ (ng/mL)	2.9	0.0–7.5	2.7	2.3–5.6	1.00
3-epi-24,25(OH)_2_D_2_ (ng/mL)	nd	nd	nd	nd	–

* *nd, not detected*.

There was no effect of D_2_ vitamer form on the concentrations of 1,25(OH)_2_D and 24,25(OH)_2_D_3_ metabolites (Table 2). Vitamin D_3_ and D_2_ forms of 1,25-dihydroxyvitamin D were identified and quantified. Median plasma concentrations of 1,25(OH)_2_D_2_ were 49% less than 1,25(OH)_2_D_3_ (*P* = 0.02).

Peaks of UV absorbance were observed coinciding with the retention times of 24,25*R*(OH)_2_D_3_ and the C-3 epimer, 3-epi-24,25*R*(OH)_2_D_3_, standards in reverse phase HPLC of extracted and fractionated plasma. Identity of the metabolites were confirmed by LC-MS/MS analysis described in the methods. No UV detectable peaks were observed during or closely following the retention time for 24*RS*,25(OH)_2_D_2_ standard. Only the vitamin D_3_ form of 24,25(OH)_2_D was detected in plasma samples of all cats. This included the alpha C-3 epimer of 24,25(OH)_2_D_3_ which was detected in six of the seven cats. Among all cats, median plasma concentrations of 24,25*R*(OH)_2_D_3_ were greater than 3-epi-24,25*R*(OH)_2_D_3_ (*P* < 0.01; Table 2).

### Effects of D_2_ Supplementation on Plasma Biochemistry and Blood Count Variables

Oral 25(OH)D_2_ compared to vitamin D_2_ supplementation was not associated with greater plasma calcium or phosphorus concentrations after 10 weeks. Additionally, there were no differences in indicators of kidney function including blood urea nitrogen, creatinine, and PTH concentrations between supplement forms [25(OH)D_2_ and vitamin D_2_] (Table 3). Clinical hematology and biochemistry values following 10 weeks of oral supplementation with 25(OH)D_2_ were not significantly different from those following vitamin D_2_ supplementation (Supplementary Tables 2, 3).

**Table 3:** Median and range of select biochemistry and endocrinology variables in seven cats after 10 weeks of oral supplementation with vitamin D_2_ or 25(OH)D_2_ while consuming a commercial feline diet containing vitamin D_3_.

		**Supplementation form**				
		**Vitamin D**_****2****_		**25(OH)D**_****2****_		
	**Reference interval**	**Median**	**Range**	**Median**	**Range**	***P*-value**
Urea nitrogen (mg/dL)	19–35	24	18–29	22	19–30	1.00
Creatinine (mg/dL)	0.9–2.0	1.4	1.0–1.7	1.5	0.9–1.8	0.69
Calcium (mg/dL)	8.6–10.4	8.9	8.6–9.8	9.2	8.9–9.5	0.44
Phosphorus (mg/dL)	2.3–5.0	4.3	3.1–4.8	4.2	3.3–4.7	0.80
Parathyroid hormone (pmol/L)	0.40–2.50	0.80	0.30–0.90	0.60	0.30–1.70	0.67
Ionized calcium (mmol/L)	1.00–1.40	1.19	1.17–1.26	1.22	1.14–1.25	0.56

## Discussion

We have described feline vitamin D metabolite formation when dietary sources of vitamin D_3_ plus vitamin D_2_ or 25(OH)D_2_ are provided. Our hypothesis that supplementation with 25(OH)D_2_ would more effectively and rapidly raise circulating 25(OH)D_2_ levels in cat plasma compared to vitamin D_2_ was supported. We report here observations of C-3 epimer formation for 25(OH)D_3_, 25(OH)D_2_, and 24,25*R*(OH)_2_D_3_. To the authors' knowledge, this is the first report confirming circulating concentrations of 1,25(OH)_2_D_2_, 24,25*R*(OH)_2_D_3_, and 3-epi-24,25*R*(OH)_2_D_3_ in the cat. We were unable to identify any circulating 24,25(OH)_2_D_2_ or 3-epi-24,25(OH)_2_D_2_ in these samples. This information may provide insight into the similarities and differences between feline vitamin D_2_ and D_3_ metabolism.

The results of this study illustrate that supplementation with vitamin D_2_ and 25(OH)D_2_ result in increased plasma concentrations of 25(OH)D_2_ and 3-epi-25(OH)D_2_ when they are provided in addition to vitamin D_3_ in the diet. During supplementation with vitamin D_2_, total 25(OH)D concentrations remained stable over time despite an increase in 25(OH)D_2_ (Table 1, Supplementary Figure 1). This could be explained by the concurrent reduction in 25(OH)D_3_ concentration. A similar effect of vitamin D_2_ supplementation on 25(OH)D_3_ levels in humans has been reported, but is not a consistent finding (56, 57). This suggests that vitamin D_2_ and vitamin D_3_ are competing substrates for 25-hydroxylase in the liver and that available 25-hydroylase activity may limit elevation of plasma 25(OH)D concentration when dietary vitamin D content is increased. An alternative explanation is that vitamin D_2_ may induce catabolism of 25(OH)D_3_, which has been theorized based on an increased ratio of 24,25(OH)D_3_ to 25(OH)D_3_ following vitamin D_2_ supplementation in humans (58). This is unlikely to be the case in cats since this pattern was not observed in our study during supplementation with 25(OH)D_2_, which resulted in an initial increase in total 25(OH)D with no change in 25(OH)D_3_ concentration (Supplementary Figure 2). The stable concentrations of plasma 25(OH)D_3_ during 25(OH)D_2_ supplementation are consistent with our knowledge of vitamin D hydroxylation in the liver, described as a relatively uncontrolled process with little negative feedback from circulating 25(OH)D concentration (59).

The ability of 25(OH)D_2_ to raise total 25(OH)D levels compared to vitamin D_2_, even when dosed at a 20% molar equivalent, indicates a greater than five times increased potency of 25(OH)D_2_ compared to vitamin D_2_. This is consistent with multiple studies and meta-analyses showing increased effectiveness of 25(OH)D_3_ compared to vitamin D_3_ in humans (24, 60, 61).

Similar to previous studies, we found that the C-3 epimer of 25(OH)D_3_ is present in cats in quantities greater than observed in other species, such as rats, dogs, and humans (11, 17, 62). There are reports of C-3 epimers of numerous vitamin D metabolites, including 1,25(OH)_2_D_3_, 1,25(OH)_2_D_2_, and 24,25(OH)_2_D_2_ which have been detected in other species and cell lines (12, 13, 15, 16, 62–67). With the methods employed here, we could not investigate occurrence of C-3 epimers of 1,25(OH)_2_D_3_ and 1,25(OH)_2_D_2_, but we did find quantifiable concentrations of 3-epi-25(OH)D_2_ and 3-epi-24,25*R*(OH)_2_D_3_ in feline plasma samples, the presence of which have not been previously reported in cats. The C-3 epimer of 25(OH)D_2_ was first detected after 2 weeks of supplementation with both D_2_ forms (Table 1). At all time points during supplementation and for both supplement forms, concentrations of 3-epi-25(OH)D_2_ were below 25(OH)D_2_. The equilibration of 3-epi-25(OH)D_2_ was much more rapid (2 weeks) than that of 25(OH)D_2_ (8 weeks). Although the role of C-3 epimerization is unknown, it has been suggested that the epimers are products of a clearance pathway for vitamin D metabolites (11). Our results are consistent with this theory, as demonstrated by increased concentrations of 3-epi-25(OH)D_2_ after initiating D_2_ vitamer supplementations. A previous report of 3-epi-25(OH)D_3_ supports C-3 epimerization as a means of protection against hypervitaminosis D by demonstrating this metabolite's ability to affect calcium homeostasis and bone mineralization (12). The presence of epimeric forms of vitamin D metabolites in high abundance may be part of the reason cats seem relatively resistant to vitamin D toxicosis even when ingesting very large amounts of vitamin D (68).

The detection of similar D_2_ and D_3_ plasma metabolites following supplementation with D_2_ forms indicates similar pathways of metabolism in cats with some possible exceptions. Firstly, we did not detect plasma concentrations of 24,25(OH)_2_D_2_ in any of the cats. It is conventionally believed that 24-hydroxylation of 25(OH)D to form 24,25(OH)_2_D is the first step in degradation of the vitamer (8). Here, the cat may be different. If the role of degradation is adopted by C-3 epimerization in cats, then we might expect plasma concentrations of 24,25(OH)_2_D to be lower in cats than plasma concentrations of 3-epi-25(OH)D. In fact, we found that 24,25*R*(OH)_2_D_3_ concentrations were similar to those of 3-epi-25(OH)D_3_ after 10 weeks. This finding seems consistent with a possible physiologic role for 24,25*R*(OH)_2_D_3_ in cats. Some investigators have found evidence that 24,25(OH)_2_D may have independent or synergistic physiological actions on bone (69). The reason we were unable to detect 24,25(OH)_2_D_2_ in plasma is unknown, though we postulate that cats may have 24-hydroxylases with lower affinity for 25(OH)D_2_ compared to 25(OH)D_3_.

Our observations of plasma 1,25(OH)_2_D concentrations may also indicate a difference between metabolism of D_2_ and D_3_ vitamers in cats. The plasma concentrations of 1,25(OH)_2_D_2_ were significantly lower than those of 1,25(OH)_2_D_3_ following 10 weeks of supplementation with both forms of vitamin D. We do not believe that the relative concentrations of the two vitamer forms of 1,25(OH)_2_D reflect a difference in substrate availability, since plasma concentrations of 25(OH)D_2_ were not significantly different from those of 25(OH)D_3_ after 10 weeks for either D_2_ supplement form (Table 1). A possible explanation is that 1α-hydroxylase in the feline kidney has a lower affinity for 25(OH)D_2_ compared to 25(OH)D_3_. Even if such an affinity difference occurs, its physiological importance is uncertain.

Of important note is that the total amount of vitamin D intake (vitamin D_3_ + D_2_ form) by the cats was well below the safe upper limit (19 μg/kg^0.67^) recommended by the National Research Council, which is based on observations for vitamin D_3_ (4). The upper limit is based in part on observations that chronic intake (18 months) of diet containing 33,840 IU/kg vitamin D by kittens and adult cats was not detrimental to reproduction, growth, or renal health (68). Nevertheless, one cat in our study was diagnosed with renal failure 11 weeks after discontinuing supplementation with 25(OH)D_2_ (0.80 μg/kg/d). After reviewing blood chemistry values for the months and years prior to the study, and based on the chronic changes detected on necropsy, we suspect this cat had an insidious progression of chronic renal disease that was not detectable on routine blood chemistry prior to study enrollment. Chronic renal failure of unknown etiology is a common cause of morbidity and mortality in domestic cats as they age (70). Although there is no evidence that renal disease was caused by supplementation with 25(OH)D_2_, this cannot be completely ruled out. However, total 25(OH)D concentrations after 10 weeks of supplementation were 44.8 ng/mL for the beta C-3 epimer and 13.1 ng/mL for the alpha C-3 epimer, which was within the range observed for the other cats supplemented with 25(OH)D_2_ (Table 1), which makes hypervitaminosis D an unlikely cause for this cat's renal disease. In addition, a second cat experienced adverse effects following supplementation with 25(OH)D_2_ for 10 weeks during the second phase of the study. This cat was diagnosed with paroxysmal third-degree AV block and persistent left bundle branch block which rapidly resolved once the supplement was discontinued. No other cats receiving 25(OH)D_2_ had cardiac abnormalities based on echocardiograms, and the relationship between these findings and supplementation are unclear.

Since use of 25(OH)D is being investigated for supplementation in multiple species, further studies to determine dose and safety of this metabolite in cats are warranted to ensure that the potent effects do not result in toxicity when supplemented at apparent physiologic doses. Ingestion of 25(OH)D in place of vitamin D has potential clinical applications for when a rapid increase in vitamin D status is sought or when absorption or metabolism of vitamin D is impaired. Although there was no difference between 25(OH)D_2_ and vitamin D_2_ on plasma hematology and biochemistry parameters, including indicators of calcium homeostasis (total calcium, phosphorus, PTH, and ionized calcium), long-term safety studies are indicated based on the adverse events observed in two of eight cats in this study. This is especially important given reports in other species that 25-hydroxyvitamin D is the more active form of vitamin D and ~2–6 times more potent than vitamin D, a feature currently being exploited in studies of human and canine vitamin D supplementation (19, 22, 23, 25, 60).

One limitation of this study was the small sample size which increased the likelihood of type-2 statistical error, especially in comparisons of outcomes between supplementation of 25(OH)D_2_ and vitamin D_2_. For the dihydroxy metabolites in particular, sample loss resulted in comparisons of only 3–4 observations. Given the low number of observations, it is possible that there is a statistical difference between the supplementation groups, but because of low power we could not detect any differences.

Additionally, differentiation between effects of the D_2_ vitamers was complicated by consumption of vitamin D_3_ in the diet. Nevertheless, our findings are relevant in practice. If cats receive diets supplemented with vitamin D_2_, some vitamin D_3_ likely would be ingested because many animal-source ingredients contain vitamin D_3_ (46). Appropriately designed studies are needed to evaluate outcome differences between D vitamers (D_2_ and D_3_) when background vitamin D status is low as in vitamin D deficiency.

In conclusion, this study reports on oral administration of D_2_ vitamers and identification of novel vitamin D metabolites that may help us to better understand vitamin D metabolism in cats. The high concentrations of C-3 epimers detected in cat plasma consistent with previous studies emphasizes the importance of considering epimeric forms of vitamin D metabolites when reporting on vitamin D status in cats, as these are present in abundant quantities and may cause under- or over-estimation of circulating vitamin D depending on methods of analysis. Further studies using 25(OH)D and vitamin D_2_ forms in cats are needed before conclusions can be made about safety and efficacy of these vitamers for supplementation, or their efficacy compared to vitamin D_3_. Nonetheless, the present research indicates that oral supplementation with 25(OH)D_2_ compared to vitamin D_2_ is more potent and more rapid in raising vitamin D status in adult cats.

## Data Availability Statement

The raw data supporting the conclusions of this article will be made available by the authors, without undue reservation.

## Ethics Statement

The animal study was reviewed and approved by Animal Care and use Committee of the University of Missouri.

## Author Contributions

CR was the principal investigator of the study and contributed to designing and implementing the study, data analyses, and writing the manuscript. RB was the co-investigator of the study and contributed to study design, implementing the study, processing and analyzing the data, and writing the manuscript. All authors contributed to the article and approved the submitted version.

## Conflict of Interest

CR was employed by the University of Missouri when this research was conducted. CR was employed by the company Hill's Pet Nutrition at the time of manuscript submission. Hill's Pet Nutrition was not involved in the conception of the study, study design, data analysis, or writing of the paper. The remaining author declares that the research was conducted in the absence of any commercial or financial relationships that could be construed as a potential conflict of interest.
