# Supplementary material for: Effects of Vitamin D2 and 25-Hydroxyvitamin D2 Supplementation on Plasma Vitamin D Epimeric Metabolites in Adult Cats
Source: Front Vet Sci. 2021 Jun 7;8:654629. doi: 10.3389/fvets.2021.654629 (PMC8215352; doi:10.3389/fvets.2021.654629)
Supplement: Supplementary file 3 [file Data_Sheet_1.docx]

Supplementary Material

**Supplementary Table S1**. Proximate analysis and ingredients^*^ for commercial dry extruded feline diet^†^ as reported by the manufacturer.

| Nutrient | Amount^‡^ (%) |
| --- | --- |
| Protein | 34.40 |
| Fat | 16.90 |
| Nitrogen-free extract | 42.10 |
| Crude fiber | 0.80 |
| Calcium | 0.98 |
| Phosphorus | 0.96 |
| Potassium | 0.77 |
| Sodium | 0.35 |
| Chloride | 0.97 |
| Magnesium | 0.06 |

^*^Ingredients: Corn gluten meal, chicken, wheat flour, brewers rice, ground yellow corn, animal fat preserved with mixed-tocopherols, egg product, sodium caseinate, phosphoric acid, calcium carbonate, potassium chloride, animal digest, salt, L-Lysine monohydrochloride, dried whey, choline chloride, dicalcium phosphate, taurine, zinc sulfate, ferrous sulfate, manganese sulfate, Vitamin E supplement, niacin, citric acid, Vitamin A supplement, calcium pantothenate , thiamine mononitrate, copper sulfate, riboflavin supplement, Vitamin B-12 supplement, pyridoxine hydrochloride, folic acid, Vitamin D-3 supplement, calcium iodate, biotin, menadione sodium bisulfite complex, sodium selenite.

^†^Purina Pro Plan FOCUS Adult Urinary Tract Health formula; 19,600 kJ of metabolizable energy per kilogram of diet dry matter

^‡^Dry matter basis; 6.98% moisture as fed.

**Supplementary Table S2**. Effect of supplementation with vitamin D_2_ or 25(OH)D_2_ on median plasma clinical biochemistry variables in adult cats (n=7).

|  |  | Supplementation Type | | | |  |
| --- | --- | --- | --- | --- | --- | --- |
|  |  | Vitamin D_2_ | | 25(OH)D_2_ | |  |
|  | Reference Interval | Median | Range | Median | Range | P-value |
| Glucose (mg/dL) | 80-155 | 80 | 73-92 | 79 | 76-90 | 0.93 |
| Urea nitrogen (mg/dL) | 19-35 | 24 | 18-29 | 22 | 19-30 | 1.00 |
| Creatinine (mg/dL) | 0.9-2.0 | 1.4 | 1.0-1.7 | 1.5 | 0.9-1.8 | 0.69 |
| Sodium (mEq/L) | 148-154 | 154 | 150-157 | 154 | 152-156 | 0.58 |
| Potassium (mEq/L) | 3.1-4.6 | 4.1 | 3.8-4.5 | 4.1 | 3.7-4.5 | 0.93 |
| Chloride (mEq/L) | 112-123 | 122 | 119-124 | 121 | 120-123 | 0.53 |
| Bicarbonate (mEq/L) | 13-23 | 10 | 8-11 | 10 | 7-13 | 0.62 |
| Anion Gap (mEq/L) | 13-26 | 27 | 25-29 | 26 | 24-29 | 0.18 |
| Albumin (g/dL) | 2.8-3.7 | 3.1 | 2.9-3.2 | 3.0 | 2.8-3.3 | 0.45 |
| Total Protein (g/dL) | 6.1-8.1 | 6.5 | 6.0-6.9 | 6.3 | 5.9-7.5 | 0.84 |
| Globulin (g/dL) | 2.5-5.2 | 3.4 | 2.8-3.9 | 3.4 | 2.9-4.2 | 0.90 |
| Calcium (mg/dL) | 8.6-10.4 | 8.9 | 8.6-9.8 | 9.2 | 8.9-9.5 | 0.44 |
| Phosphorus (mg/dL) | 2.3-5.0 | 4.3 | 3.1-4.8 | 4.2 | 3.3-4.7 | 0.80 |
| Cholesterol (mg/dL) | 80-258 | 170 | 146-211 | 158 | 126-218 | 0.75 |
| Total Bilirubin (mg/dL) | 0.1-0.2 | 0.1 | 0.1-0.1 | 0.1 | 0.1-0.1 | - |
| ALT (IU/L)^*^ | 28-84 | 41 | 28-71 | 33 | 24-75 | 0.93 |
| ALP (IU/L) | 13-50 | 14 | 10-20 | 14 | 11-21 | 0.83 |
| GGT (IU/L) | 0-3 | < 3 | <3-<3 | < 3 | <3-<3 | - |
| CK (IU/L) | 65-574 | 99 | 49-427 | 124 | 58-169 | 1.00 |

ALT = alanine amino transferase. ALP = alkaline phosphatase. GGT = gamma-glutamyl transferase. CK = creatine kinase.

*International units per liter.

**Supplementary Table S3**. Effect of supplementation with vitamin D_2_ or 25(OH)D_2_ on median plasma clinical hematology variables in adult cats (n=7).

|  |  | Supplementation Type | | | |  |
| --- | --- | --- | --- | --- | --- | --- |
|  |  | Vitamin D_2_ | | 25(OH)D_2_ | |  |
|  | Reference Interval | Median | Range | Median | Range | P-value |
| Plasma Protein (g/dL) | 6.3-8.5 | 7.0 | 6.7-7.7 | 6.8 | 6.7-8.0 | 1.00 |
| WBC (×1,000/µl) | 3.82-13.14 | 10.42 | 4.26-14.63 | 9.49 | 4.14-12.01 | 0.82 |
| RBC (× 1,000,000/µl) | 6.65-11.06 | 8.29 | 8.04-10.34 | 8.58 | 7.49-9.89 | 1.00 |
| Hemoglobin (g/dL) | 9.7-16.1 | 11.8 | 10.9-12.7 | 11.8 | 10.2-12.4 | 0.97 |
| Hematocrit (%) | 28.0-48.1 | 34.5 | 32.0-36.4 | 35 | 31-37 | 0.95 |
| MCV (fL) | 36.3-51.1 | 39.7 | 35.2-44.0 | 39.0 | 36.0-45.1 | 0.77 |
| MCH (pg) | 12.4-17.6 | 13.5 | 12.3-14.6 | 13.6 | 12.3-14.5 | 0.98 |
| MCHC (g/dL) | 31.2-36.8 | 34.5 | 31.9-35.0 | 33.7 | 32.2-35.5 | 0.72 |
| Platelets (× 1,000/µl) | 166-466 | 339 | 260-448 | 365 | 270-513 | 0.58 |
| Seg. Neutrophils (× 1,000/µl) | 2.27-9.82 | 5.589 | 2.684-10.68 | 6.36 | 2.36-8.767 | 0.89 |
| Band Neutrophils (× 1,000/µl) | 0.00-0.20 | 0 | 0-0 | 0 | 0-0 | - |
| Lymphocytes (× 1,000/µl) | 0.76-5.63 | 1.747 | 0.72-4.495 | 2.26 | 0.395-3.094 | 0.56 |
| Monocytes (× 1,000/µl) | 0.00-0.45 | 0.15 | 0.1-0.238 | 0.12 | 0.056-0.47 | 0.90 |
| Eosinophils (× 1,000/µl) | 0.00-1.88 | 0.88 | 0.256-1.701 | 0.66 | 0.238-1.03 | 0.18 |
| Basophils (× 1,000/µl) | 0.00-0.16 | 0 | 0-0 | 0 | 0-0.119 | 0.62 |

WBC = white blood cell count. RBC = red blood cell count. MCV = mean corpuscular volume. MCH = mean corpuscular hemoglobin. MCHC = mean corpuscular hemoglobin concentration.
